# Influence of Factors in the Photopolymerization Process on Dental Composites Microhardness

**DOI:** 10.3390/ma15186459

**Published:** 2022-09-17

**Authors:** Jordan Maximov, Tsanka Dikova, Galya Duncheva, Georgi Georgiev

**Affiliations:** 1 Faculty of Mechanical Engineering, Technical University of Gabrovo, 4 Hadji Dimitar Str., 5300 Gabrovo, Bulgaria; 2 Faculty of Dental Medicine, Medical University of Varna, 84 Tsar Osvoboditel Blvd., 9000 Varna, Bulgaria

**Keywords:** light-cured dental composites, microhardness, light intensity, irradiation time, layer thickness

## Abstract

The aim of the present paper is to investigate the influence of factors in photopolymerization process that govern microhardness of three types of dental composites—universal (UC), bulk-fill (BC), and flowable (FC). Cylindrical specimens with different thicknesses are made and light cured. The significance of light intensity, irradiation time, and layer thickness on Vickers microhardness is evaluated by experimental design, analysis of variance, and regression analysis. It is found that the main factor influencing the microhardness on the top surface of the three composites is light intensity. The second factor is layer thickness for the UC and FC, while for BC, it is curing time. The third factor is curing time for the first two composites and layer thickness for bulk-fill. The significance of factors’ influence on the microhardness of the bottom surface is the same for the UC and FC, but different for BC. The main factor for the first two composites is layer thickness, followed by curing time and light intensity. For bulk-fill, curing time is main factor, light intensity is second, and layer thickness is last. Different significance of factors influencing the microhardness on top and bottom surfaces of investigated composites is revealed for the first time in the present study.

## 1. Introduction

Resin-based composites (RBCs), also known as dental composites, are widely used in restorative dentistry due to their many advantages, such as excellent esthetics, conservation of tooth structure, good longevity, affordable price, and possibility for repair [1]. They are used in dental practices as restorative materials, sealants, liners, veneers, crowns, cements, etc. [2]. The main components of RBC include organic matrix, inorganic filler, coupling agents, and initiators of the polymerization process [3,4,5]. The organic resin matrix phase is made from a mixture of multifunctional monomers (Bis-GMA, UDMA, TEGDMA, etc.), while the filler phase contains micro/nano-sized fillers, which are mainly used as a reinforcement [6,7].

Depending on their viscosity, composites can be divided into compactable and flowable. The latter have a reduced filler content (37–53 vol. %), which gives them a low viscosity with a flowable character, spreading uniformly, and adapting intimately to the prepared tooth surfaces. The main advantages of flowable over compactable composites are easier handling properties during manipulation, better adaptation to the tooth surface, and higher flexibility [8]. Of course, what makes flowable composites so effective for some restorations also limits their effectiveness in others. The lower filler content of these materials can lead to more polymerization shrinkage. Moreover, less filler leads to a reduction in the composite’s mechanical properties [9]. However, not all flowables have the same composition. Newer materials have higher filler contents than their predecessors, which improves some of the well-known disadvantages and gives them higher strength and wear resistance. These improved materials can be used for Class I, II, III, IV, and V restorations, and with similar results to more viscous composites [10].

Conventional RBCs are applied using an incremental technique, which is a time-consuming process and contributes to greater inaccuracies. To simplify the procedure, the manufacturers create bulk-fill composites that allow light curing layers of up to 5 mm, while ensuring sufficient depth of cure. These materials can be divided into two groups: Base and full-body bulk-fill composites. Base bulk-fill composites have a low viscosity (they are flowable), which enables placement and adaptation in less accessible cavities. These composites have a lower filler content and, respectively, lower wear resistance, hence, capping with a conventional composite is required [11]. The full-body bulk-fill composites are highly viscous because of their higher filler loads and allow the whole restoration to be placed at once without requiring any coverage. The application of bulk fill composites in posterior restorations reduces cusp deflection [12,13,14] and polymerization stress [15], thus increasing the fracture resistance of the restoration and hard dental tissues [12].

The degree of conversion achieved within a composite is important because it controls several properties of the cured material, including mechanical strength, polymerization shrinkage, wear behavior, and monomer release [16]. The degree of conversion depends on many factors: Light intensity, curing time, layer thickness, distance and angulation of the light curing tip to the irradiated surface, color of the composite, type and amount of filler, and material temperature [17].

Many in vitro studies have been conducted over the years to show how different irradiation times and light intensities affect the mechanical properties of the restoration [18,19,20]. They all confirm that the amount of light energy needed for the proper polymerization of the restoration can be achieved by many combinations between light intensity and irradiation time, and the higher the intensity of the light curing unit, the shorter the duration of the polymerization. The abovementioned studies indicate that increasing the light curing time results in higher overall radiant exposure reaching the composite layer. Therefore, better polymerization can be obtained, especially with a thick composite layer or a light curing unit with low light intensity.

Another factor of fundamental importance for the proper polymerization of the RBC restoration is the layer thickness. Many authors in their studies have reported that for conventional composite, a layer thickness of 2 mm is optimal when the incremental technique is applied [13,21]. Placing increments thicker than 2–2.5 mm significantly reduces the mechanical and physical properties of the restoration and, respectively, its longevity [17,22]. The use of bulk-fill composites allows adequate polymerization for thicker layers up to 5 mm.

The successful polymerization of RBCs can be evaluated by their hardness. There is a positive correlation between the conversion ratio and hardness of dental composites [23,24]. It was found that an 80% bottom-to-top hardness ratio corresponds to a 90% conversion ratio [25]. On the other hand, the wear and fracture resistance, as well as the durability of the restoration, are defined by the composite hardness. 

The present study aims to investigate the influence of factors in the photopolymerization process (light intensity, irradiation time, and layer thickness) on the microhardness of three types of dental composites (conventional, bulk-fill, and flowable). The evaluation of the factors is performed by dispersion and regression analyses. The combination of these methods gives the opportunity to study the influence of each factor on the hardness and also to determine the significance of the different factors. To the best of our knowledge, different significance of the factors influencing the microhardness on the top and bottom surfaces of the investigated composites is established for the first time in the present study. 

## 2. Materials and Methods

### 2.1. Materials and Specimen Preparation

Three types of light-cured resin-based composites were used in the research: Universal nanohybrid composite (UC) (Evetric, Ivoclar Vivadent, Liechtenstein), nanohybrid bulk-fill composite (BC) for posterior restorations (Filtek One Bulk Fill Restorative, 3M, Saint Paul, MN, USA), and universal nanofilled flowable composite (FC) with high filler content (G-aenial Universal Flo, GC, Fuchu, Japan) with composition shown in Table 1. Round samples (5 mm diameter) were made of the three composites with the same A2 shade. The samples were manufactured in polyurethane molds with thicknesses of 2, 3, and 4 mm. The polymerization was carried out by a light-curing unit (LCU) (Curing Pen, Eighteeth, Changzhou Sifary Medical Technology Co., Ltd., Changzhou, China), varying the light intensity (600, 1000, and 1500 mW/cm^2^) and irradiation time (20, 40, and 60 s). More detailed information about sample preparation was given in our previous works [26,27].

Three specimens were manufactured for each combination of parameters. According to the experimental design, shown in Table 2, fourteen different combinations of parameters were used for each composite type. Therefore, the specimens were divided into 3 groups depending on the composite type—UC, BC, and FC. Each group was divided into 14 subgropus (3 samples in each) according to the photopolymerization parameters. Totally, 126 specimens were prepared for the research, or 42 for each composite. After sample polymerization, they were stored for 24 h at room temperature in a dry, dark container before hardness measurements were made.

### 2.2. Microhardness Measurements

The surface microhardness measurements on Vickers method ($HV_{0.05}$) were made using a ZHVµ Zwick/Roell microhardness tester with computerized processing of the measurement results, using a 0.05 kgf load and a 10 s holding time. Five measurements were made for the top and bottom surface of each specimen. The final value of the top and bottom surface microhardness corresponded to the grouping center. 

### 2.3. Experimental Design

The governing factors and their levels are shown in Table 3. Physical coordinates ${\tilde{x}}_{i},\;i=1,2,3$ are transformed into dimensionless coordinates $x_{i},\;i=1,2,3$ by the formula

(1) $$x_{i}=\left({\tilde{x}}_{i}-{\tilde{x}}_{0,i}\right)/\lambda_{i},$$

where $\lambda_{i}=\left({\tilde{x}}_{\max,i}-{\tilde{x}}_{\min,i}\right)/2$, ${\tilde{x}}_{0,i}$, ${\tilde{x}}_{\max,i}$ and ${\tilde{x}}_{\min,i}$ are, respectively, average, upper and lower levels of the $i_{th}$ physical factor. The objective functions are $Y_{1}$ and $Y_{2}$, surface microhardness respectively on the top and bottom surface of the composite layer. An optimal second-order composition plan was chosen (Table 2).

Analysis of variance (ANOVA)—two-way variant and regression analysis were conducted in order to study the factor influence, using QstatLab software (V. 5.5, creators Vuchkov I. N. and Vuchkov I. I., Sofia, Bulgaria). The objective functions in the regression models were chosen to be polynomials from the second order, given the number of levels of the governing factors:

(2) $$Y_{i}=b_{0}+\sum_{j=1}^{3}b_{j}x_{j}+\sum_{j=1}^{2}\sum_{k=j-1}^{3}b_{jk}x_{j}x_{k}+\sum_{j=1}^{3}b_{jj}x_{j}^{2},\;i=1,\;2$$

The absolute value of the coefficients in the objective functions (2) shows the significance of the respective factor. 

## 3. Results

The results we obtained show that the highest microhardness was in a sample of BC Filtek One Bulk Fill Restorative: 57.9–69.1 HV on the top surface and 45.4–67.5 HV on the bottom, depending on the curing modes used (Table 3). It is followed by FC G-aenial Universal Flo with hardness 42.4–51.1 HV and 13.1–47.7 HV on the top and bottom surfaces, respectively. The lowest hardness was observed in UC Evetric, with values of hardness on the top surface of 42.0–62.7 HV and 12.2–51.3 HV on the bottom. Regardless of the curing modes used, the smallest differences between the hardness of the two surfaces were observed in BC Filtek One Bulk Fill Restorative, and the largest—in UC Evetric. 

The polynomial coefficients of the objective functions are shown in Table 4.

### 3.1. UC Evetric

**Figure 1:**
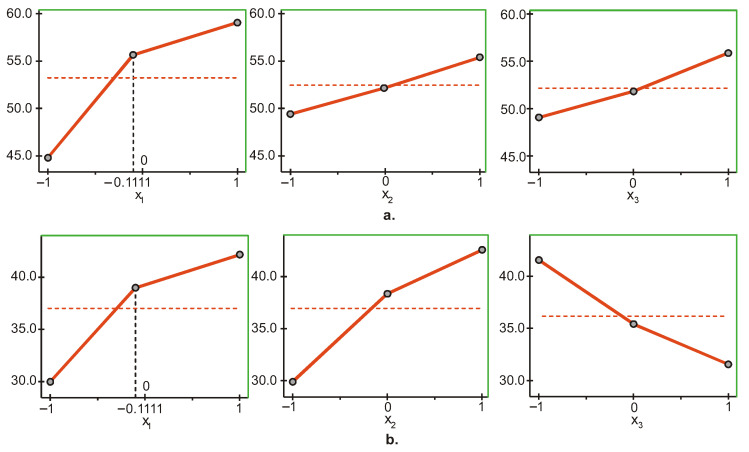
Influence of the different factors of photopolymerization on the microhardness on: (**a**) The top and (**b**) bottom surfaces of UC Evetric ($x_{1}$—light intensity, $x_{2}$—curing time, $x_{3}$—layer thickness). Red dashed line: average value of micro-hardness; black dashed line: the actual coded value of $x_{1}$.

The most significant factor influencing the surface hardness $Y_{1}$ of UC Evetric is light intensity ($x_{1}$), followed by layer thickness ($x_{3}$) and irradiation time ($x_{2}$) (Figure 1a). The upper levels of the three factors ($x_{1}$,$x_{2}$,$x_{3}$) maximize the objective function $Y_{1}$, and the lower levels minimize it. The maximum/minimum of the objective function $Y_{1}$ is reached when the three factors are maintained at the upper/lower level. This shows that the maximum hardness on the top surface is obtained at maximum values of the three parameters intensity, time, and layer thickness (1500 mW/cm^2^, 60 s and 4 mm), and the minimum hardness at minimum values of the parameters (600 mW/cm^2^, 20 s and 2 mm).

The influence of the different factors on the hardness of the bottom surface $Y_{2}$ of UC Evetric is presented in Figure 1b. The graphs show that the most significant factor, in this case, is layer thickness ($x_{3}$), followed by irradiation time ($x_{2}$) and light intensity ($x_{1}$). The difference in the significance of the last two factors is very small, and a regression model in dimensionless coordinates is needed for a more accurate estimate. The maximum/minimum of the objective function $Y_{2}$ is achieved by maintaining the first two factors ($x_{1}$ and $x_{2}$) at the upper/lower level and the third factor ($x_{3}$) at the lower/upper level. Therefore, maximum/minimum hardness on the bottom surface is obtained at maximum/minimum values of the first two parameters, intensity and time (1500 mW/cm^2^ and 60 s/600 mW/cm^2^ and 20 s), and the values of the third parameter, layer thickness, are respectively minimum/maximum (2 mm/4 mm).

**Figure 2:**
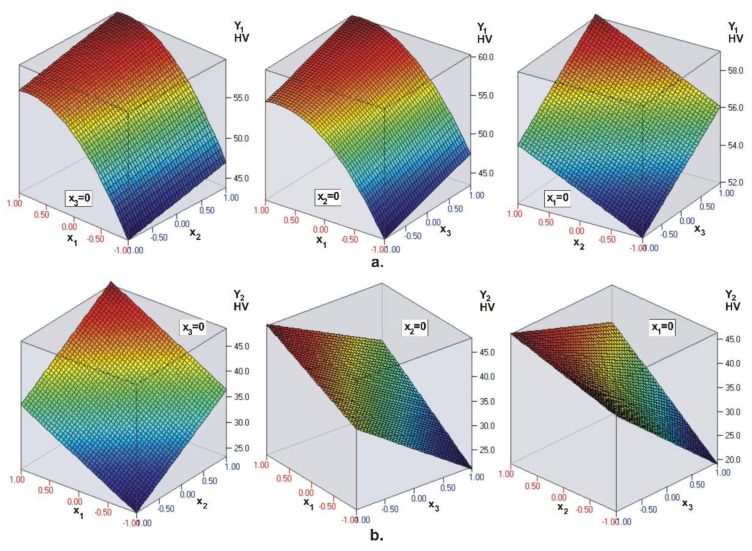
UC Evetric. Visualization of the regression models for: (**a**) Top surface microhardness and (**b**) bottom surface microhardness, $Y_{2}$ of the composite layer ($x_{1}$—light intensity, $x_{2}$—curing time, $x_{3}$—layer thickness). The dark blue color corresponds to the minimum micro-hardness values, and the deep red color corresponds to the maximum values.

The coefficients in the objective functions (see Table 4) confirm the conclusions obtained via ANOVA (Figure 1). The visualization of the regression models for the hardness on the top surface ($Y_{1}$) of the composite layer and hardness on the bottom surface ($Y_{2}$) is presented in Figure 2. The coefficients in the regression model $Y_{1}$ confirm that light intensity ($x_{1}$) is the most significant factor, followed by layer thickness ($x_{3}$) and irradiation time ($x_{2}$). From the regression model $Y_{2}$ it is clear that the most significant factor is layer thickness ($x_{3}$), followed by irradiation time ($x_{2}$) and light intensity ($x_{1}$). Therefore, the intensity of the LCU has the greatest influence on the top surface hardness of the UC Evetric restoration, and as the intensity increases, so does the hardness. Layer thickness has the strongest effect on the hardness of the bottom surface, but here the dependence is reversed: Increasing the layer thickness leads to a decrease in hardness.

### 3.2. BC Filtek One Bulk Fill Restorative

**Figure 3:**
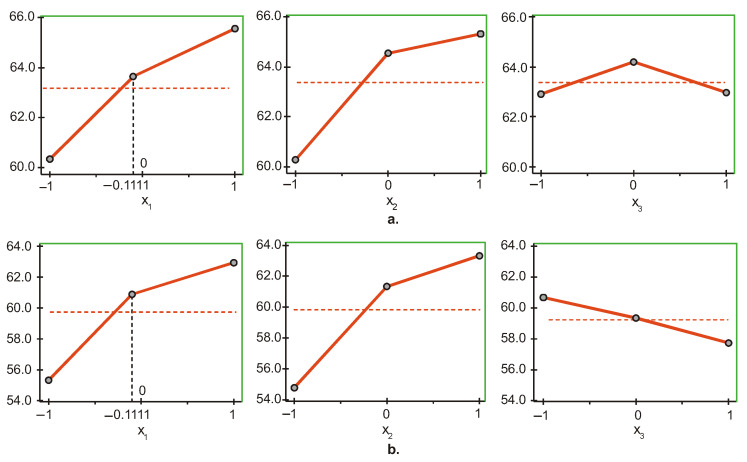
Influence of the different factors of photopolymerization on the microhardness on: (**a**) The top, and (**b**) bottom surfaces of BC Filtek One Bulk Fill Restorative ($x_{1}$—light intensity, $x_{2}$—curing time, $x_{3}$—layer thickness). Red dashed line: average value of micro-hardness; black dashed line: the actual coded value of $x_{1}$.

The graphs in Figure 3a show that the most significant factor influencing the hardness of the top surface $Y_{1}$ of BC Filtek One Bulk Fill Restorative is light intensity ($x_{1}$), followed by irradiation time ($x_{2}$) and layer thickness ($x_{3}$). The upper levels of the first two factors ($x_{1}$ and $x_{2}$) and the medium level of the third factor ($x_{3}$) maximize $Y_{1}$, and the lower levels minimize the objective function. This means that the maximum hardness on the top surface is obtained when the light intensity and irradiation time have the highest values (1500 mW/cm^2^ and 60 s) at a medium layer thickness of 3 mm, whereas working with the minimum values of the three factors (600 mW/cm^2^, 20 s and 2 mm) ensures minimum hardness on the top surface.

At the bottom surface hardness $Y_{2}$ (Figure 3b), however, the most significant factor is time ($x_{2}$), followed by intensity ($x_{1}$) and thickness ($x_{3}$). The maximum/minimum of $Y_{2}$ is achieved by maintaining the first two factors ($x_{1}$ and $x_{2}$) at the upper/lower level and the third factor ($x_{3}$) at the lower/upper level. Therefore, maximum/minimum hardness on the bottom surface is obtained at maximum/minimum values of intensity and time (1500 mW/cm^2^ and 60 s/600 mW/cm^2^ and 20 s) and at minimum/maximum layer thickness, respectively, 2 mm/4 mm.

**Figure 4:**
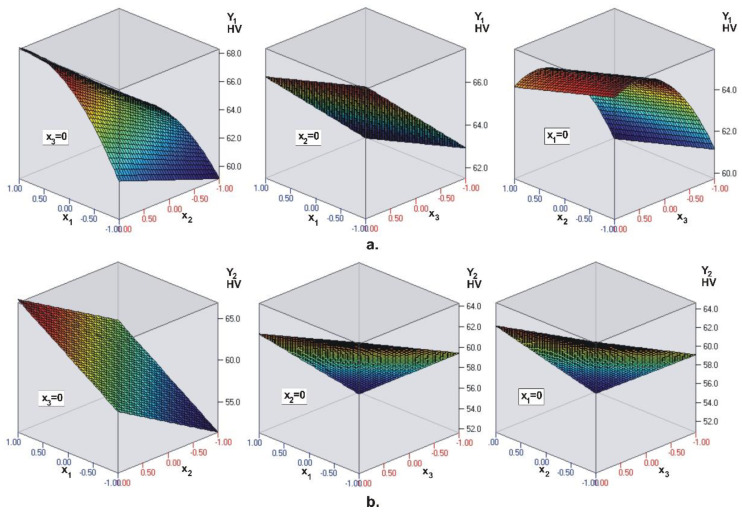
BC Filtek One Bulk Fill Restorative. Visualization of the regression models for: (**a**) Top surface microhardness $Y_{1}$ and (**b**) bottom surface microhardness $Y_{2}$, of the composite layer ($x_{1}$—light intensity, $x_{2}$—curing time, $x_{3}$—layer thickness). The dark blue color corresponds to the minimum micro-hardness values, and the deep red color corresponds to the maximum values.

Similar to UC Evetric, the coefficients (see Table 4) in front of the dimensionless coordinates confirm the conclusions of ANOVA. The visualization of the regression models for hardness on the top surface $Y_{1}$ of the composite layer and hardness on the bottom surface $Y_{2}$ is shown in Figure 4. The regression model $Y_{1}$ for surface hardness of BC Filtek One Bulk Fill Restorative confirms that the most significant factor is light intensity ($x_{1}$), followed by irradiation time ($x_{2}$) and layer thickness ($x_{3}$). For the bottom surface hardness $Y_{2}$, the most significant factor is time ($x_{2}$), followed by intensity ($x_{1}$) and thickness ($x_{3}$). Therefore, for BC Filtek One Bulk Fill Restorative, the intensity of the LCU has the greatest influence on the hardness of the top surface $Y_{1}$, and the irradiation time on the hardness of the bottom surface $Y_{2}$. Here, too, intensity and time have a positive effect: With their increase, the hardness on both surfaces increases. The layer thickness has a negative effect on the hardness of the bottom surface: Thicker composite layers have lower bottom surface hardness.

### 3.3. FC G-aenial Universal Flo

**Figure 5:**
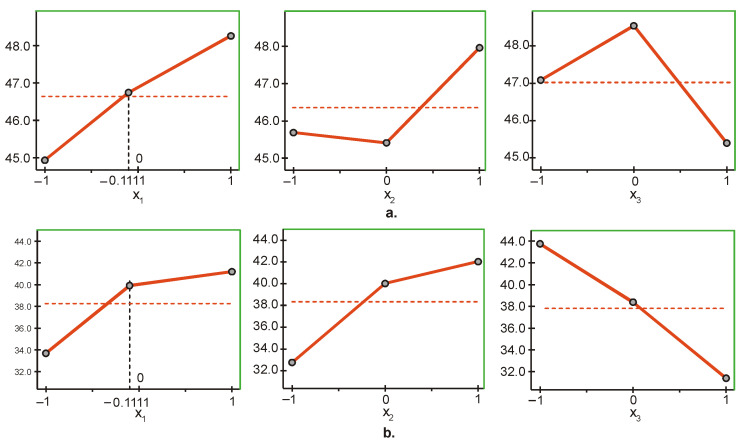
Influence of the different factors of photopolymerization on the microhardness on: (**a**) The top, and (**b**) bottom surfaces of FC G-aenial Universal Flo ($x_{1}$—light intensity, $x_{2}$—curing time, $x_{3}$—layer thickness). Red dashed line: average value of micro-hardness; black dashed line: the actual coded value of $x_{1}$.

The graphs in Figure 5a for FC G-aenial Universal Flo, similar to UC Evetric, show that the most significant factor for top surface hardness $Y_{1}$ is intensity ($x_{1}$), followed by layer thickness ($x_{3}$) and time ($x_{2}$). In contrast, different factors have different effects on the objective function. The upper levels of the first two factors ($x_{1}$ and $x_{2}$) and the middle level of the third factor ($x_{3}$) maximize $Y_{1}$. The lower level of the first factor ($x_{1}$), the middle level of the second factor ($x_{2}$) and the upper level of the third factor ($x_{3}$) minimize the objective function $Y_{1}$. Therefore, the maximum hardness on the top surface is obtained at maximum values of the intensity and irradiation time (1500 mW/cm^2^ and 60 s) and at middle values of the layer thickness of 3 mm. The minimum hardness is obtained by working with a minimum intensity of 600 mW/cm^2^, an irradiation time of 40 s, and a maximum layer thickness of 4 mm.

In Figure 5b it is shown that for the hardness on the bottom surface $Y_{2}$ the most important factor is layer thickness ($x_{3}$), followed by irradiation time ($x_{2}$) and light intensity ($x_{1}$). The difference in the significance is very small, and a regression model in dimensionless coordinates was used for a more accurate estimate. The maximum/minimum of $Y_{2}$ is achieved by maintaining the first two factors ($x_{1}$ and $x_{2}$) at the upper/lower level and the third factor ($x_{3}$) at the lower/upper level. Here, as with UC Evetric, maximum/minimum hardness on the bottom surface is obtained at maximum/minimum values of the first two parameters, intensity and time (1500 mW/cm^2^ and 60 s/600 mW/cm^2^ and 20 s), while the values of the third parameter, layer thickness, are minimum/maximum (2 mm/4 mm).

**Figure 6:**
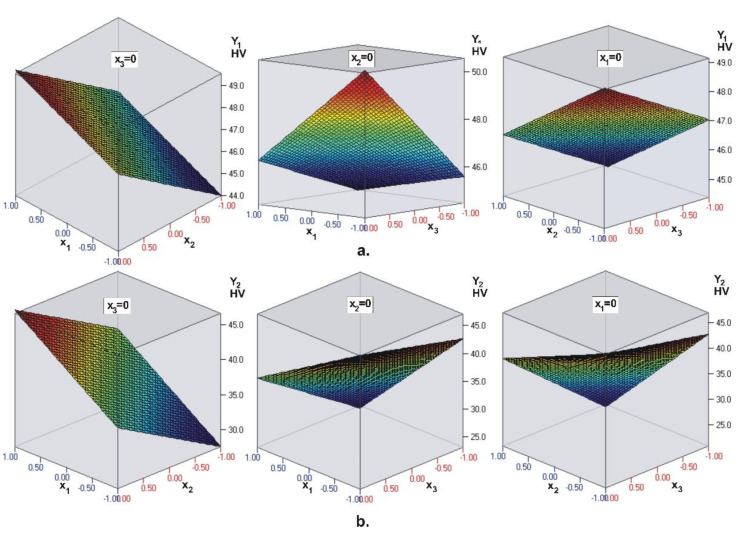
FC G-aenial Universal Flo. Visualization of the regression models for: (**a**) Top surface microhardness $Y_{1}$ and (**b**) bottom surface microhardness $Y_{2}$, of the composite layer ($x_{1}$—light intensity, $x_{2}$—curing time, $x_{3}$—layer thickness). The dark blue color corresponds to the minimum micro-hardness values, and the deep red color corresponds to the maximum values.

Similar to the previous two composites, the coefficients (see Table 4) of the dimensionless coordinates confirm the conclusions of ANOVA. The visualization of the regression models for top surface hardness $Y_{1}$ of the composite layer and bottom surface hardness $Y_{2}$ is shown in Figure 6. The regression model for the objective function $Y_{1}$ (hardness on the top surface) confirms that the most significant factor is the intensity ($x_{1}$), followed by the thickness ($x_{3}$) and time ($x_{2}$).

The regression model for the objective function *Y*_2_ (hardness on the bottom surface) shows that the most important factor is layer thickness ($x_{3}$), followed by irradiation time ($x_{2}$) and light intensity (.$x_{1}$.). Therefore, similar to UC Evetric, in FC G-aenial Universal Flo the intensity of the LCU has the greatest influence on the hardness on the top surface $Y_{1}$, and the layer thickness has the strongest effect on the hardness on the bottom surface $Y_{2}$.

## 4. Discussion

Many factors influence the photopolymerization process of resin-based composites. They can be divided into two main groups: (1) External factors relating to the light-curing parameters and (2) internal ones, including the RBC compositions. The light-curing unit used, light intensity and spectrum, light tip position and distance from the composite surface, curing depth, and irradiation time belong to the first group. The co-monomer composition and ratio, photoinitiator type, type of fillers, and volume fraction, as well as their shapes and sizes, are part of the second group [32,33,34,35,36]. Among them, the composition of the RBC: Its monomer system, filler amount, and the filler-matrix interface, are the most important factors influencing the mechanical properties and microhardness [28,32,34,35,37]. The strength characteristics and hardness increase mainly with an increase in the filler quantity [4,38,39]. The composition of the fillers, their shapes, and sizes have an indirect influence, raising the filler volume fraction by varying shape, size, and distribution of the fille particles [34].

In our study, the highest microhardness on both surfaces is established for the BC Filtek One Bulk Fill Restorative, followed by the flowable composite G-aenial Universal Flo and the universal nano-hybrid Evetric. Their filler content is as follows: 76.5%, 69% and 80–81% (Table 1). According to the previous comments, the hybrid composite with the highest filler content should possess the highest microhardness, i.e., UC Evetric. However, in our study, this composite is characterized by having the lowest microhardness due to particles with lower hardness in its composition: Prepolymers and mixed oxides. The fillers of the BC Filtek One Bulk Fill Restorative and FC G-aenial Universal Flo consist mainly of particles with high hardness (ceramic, silica, zirconia, and strontium glass) that define their higher microhardness.

The depth to which the composite hardens in the light-curing process is referred to as the depth of cure [33]. If the bottom-to-top hardness ratio is equal to 0.80 or higher, the depth of cure is considered adequate [35,39]. The depth of cure depends on the fillers’ composition, shade, and translucency as well as the light intensity and distance from the curing tip [33]. On the other hand, the depth of cure is defined by the degree of conversion of the composite matrix. Hence, the microhardness can also be considered as an indicator of the degree of conversion [37].

The degree of conversion depends mainly on the light penetration in the composite material, which is influenced by the filler-organic matrix system [36]. The translucency of resin-based composites can be increased by reduction of the filler amount, usage of filler with larger sizes, and investigation of the refractive index of the fillers-organic matrix system [39]. The use of fillers with larger sizes decreases the total filler-matrix interface, thus reducing the scattered light and enhancing the transmission of the curing light [35,36].

Light transmission is characterized by the light attenuation coefficient and refractive index. The light attenuation through the composite thickness is a result of the complex process of scattering and absorption of light by the composite’s components: Monomers, pigments, and fillers [40]. In RBCs, the light intensity scattering depends on the fillers’ volume fraction, filler size, and refractive index of the monomers and fillers. There is a positive linear correlation between the attenuation coefficient and the filler volume: decreasing the volume fraction leads to low light attenuation. The refractive index is a measure of the speed of light in the material [40]. As RBCs are mixtures of different monomers and fillers, characterized by their own refractive indexes, it is highly difficult to define the resultant refractive index of the composite. It is found that the refractive index on the top surface is high due to the higher condensation due to high light intensity, while that on the bottom surface is lower with 0.17–0.55% for different composites due to the lower condensation with decreased light intensity [40]. The bulk-fill composites are characterized by a low attenuation coefficient compared to conventional RBCs, defining high light transmission through the material. Changes in refractive index and microhardness on the bottom surface of bulk-fill composites are low because of the low light attenuation.

In the nanofilled composites, the sizes of the particles vary between 0.005 and 0.01 µm [33,41]. The particles in this nano-range cannot react with visible light, and there is no scattering. As a result, significant absorption of the light occurs through the thickness of the composite, leading to a higher depth of cure and modulus of elasticity.

The viscosity of the monomers and the flexibility of their structure are other factors influencing the degree of conversion [42]. The established degree of conversion of the various monomers used in resin-based composites is in the following order: Bis-GMA < Bis-EMA < UDMA < TEGDMA [43]. Due to the lower viscosity of UDMA compared to Bis-GMA, the bulk-fill composites containing UDMA and TEGMA are characterized by having the highest degree of conversion [44].

With the use of dispersion and regression analyses, the present study establishes the influence of light intensity, irradiation time, and layer thickness on the hardness of three types of dental composites. These methods, on the one hand, show the influence of each factor on the hardness. On the other hand, they make it possible to determine the significance of the different factors.

Our study has shown that the main factor influencing the microhardness on the top surface of the three composites is the light intensity (Table 5). As the composites with the same A2 shade are used, and the distance between the top surface and the LCU tip is the same, the significance of this factor is defined by the high intensity of the curing light [40]. The second factor affecting the top surface microhardness is the layer thickness for the UC Evetric and FC G-aenial Universal Flo, while for the BC Filtek One Bulk Fill Restorative it is curing time. The third factor is the curing time for the first two composites and layer thickness for the bulk-fill.

The significance of the factors’ influence on the microhardness of the bottom surface is the same for the UC Evetric and FC G-aenial Universal Flo, but different for the BC Filtek One Bulk Fill Restorative. The main factor for the first two composites is layer thickness, followed by curing time and light intensity (Table 5). For the BC Filtek One Bulk Fill Restorative, the main factor is the curing time. The second is light intensity, and layer thickness is last.

The difference in the significance of the factors influencing the microhardness on the top and bottom surfaces of the investigated composites is defined mainly by their composition: The monomers of the resin matrix, filler type, amount, and sizes.

The UC Evetric is a typical representative of nano-hybrid universal composites with a high filler content of 80–81% and filler sizes ranging from 40 nm to 3 μm (Table 1). Its organic matrix consists mainly of UDMA, but also of 3–10% Bis-GMA and 3–10% Bis-EMA. The last two monomers are characterized by a lower degree of conversion [43]. The highest volume fraction of the filler and the particle sizes, larger than the wavelength of the visible light, will cause scattering of the curing light and high attenuation coefficient. These circumstances will make it difficult to harden a thick layer of UC Evetric as the lower degree of conversion of Bis-GMA and Bis-EMA will have additional negative effects. That is the reason the layer thickness has a decisive role in the microhardness on the bottom surface of this composite (Table 3) and is the second-most significant factor affecting the microhardness on the top surface.

The organic matrix of the BC Filtek One Bulk Fill Restorative consists mainly of UDMA and AUDMA (Table 1), characterized by having the highest degrees of conversion [44]. The nanometer size of the fillers (from 4–20 nm of the particles up to 100 nm of the agglomerates) defines a low attenuation coefficient and high light transmission [33,40], resulting in a minimal difference between the microhardness of the top and bottom surfaces. Due to the high penetration of light through the material, the layer thickness has the lowest impact on the microhardness on the top and bottom surfaces of the bulk-fill composite (Table 3), and the curing time is the decisive factor for the microhardness on the bottom surface.

The presence of the low viscosity monomers UDMA, TEGDMA, and 5–10% Bis-EMA in the composition, the lower volume fraction of filler of 69%, and filler particles with sizes between 16 and 200 nm (Table 1) define the low viscosity of the flowable composite G-aenial Universal Flo. According to the producer [30], new glass particles are adopted as filler: Ultra-fine strontium glass that possesses superior translucency and low refractive index. The filler particles are treated by a new silane surface treatment and are homogeneously distributed in the organic matrix, thus providing the high strength of the composite. Similar to UC Evetric, the FC G-aenial Universal Flo has monomer Bis-EMA with a lower degree of conversion in its organic matrix. The filler particle sizes are larger than those of the BC Filtek One Bulk Fill Restorative, but smaller than the UC Evetric. Therefore, the characteristics of light penetration in this composite would occupy an intermediate place between those of the two other investigated materials. Taking into account the presence of a monomer with a lower degree of conversion and particles with larger sizes, higher scattering of light could be expected compared to the bulk-fill composite. This means the layer thickness is the most significant factor influencing the microhardness on the bottom surface of the FC G-aenial Universal Flo.

In the present study, three types of dental composites are polymerized by 14 different modes varying with three parameters (light intensity, curing time, and layer thickness). They are chosen on principle of investigating one typical representative of each group (conventional, bulk-fill, and flowable). Taking into account that there is a great variety of dental composites and light curing units on the market nowadays, the limitations of this study are that only three composites are used that are polymerized with one light curing unit.

## 5. Conclusions

The influence of light intensity, irradiation time, and layer thickness on the micro-hardness of three types of dental composites (conventional, bulk-fill, and flowable) has been established in the present work. The impact and significance of the factors are evaluated by dispersion and regression analyses.

It is found that the main factor influencing the microhardness on the top surface of the three composites is the light intensity. The second factor affecting the top surface microhardness is the layer thickness for the conventional and flowable composites, while for the bulk-fill, it is curing time. The third factor is the curing time for the first two composites and layer thickness for the bulk-fill.

Our study has shown that the significance of the factors influencing the microhardness on the bottom surface are the same for the conventional and flowable composites but different for the bulk-fill. The main factor for the first two composites is the layer thickness, followed by curing time and light intensity. For the bulk-fill, curing time is the main factor, light intensity is the second, and layer thickness is the last.

The different significance of the factors influencing the microhardness on the top and bottom surfaces of the investigated composites is defined mainly by their composition: The monomers of the resin matrix, filler type, amount, and sizes.

## Figures and Tables

**Table 1:** Composition of the investigated dental composites [26].

No.	Composite	Composition		Matrix/Filler Ratio, wt %
		Component	Amount	
1	UC	Matrix: UDMA (Urethane dimethacrylate) Bis-GMA (Bisphenol A glycydil dimethacrylate) Bis-EMA (Bisphenol A polyethethylene glycol dimethacrylate) Fillers: Barium glass, ytterbium fluoride (YbF_3_), mixed oxides, and prepolymers 40 nm–3 μm.	10–25%; 3–10%; 3–10%	19–20/80–81
	Evetric			
	[28]			
2	BC Filtek One Bulk Fill Restorative [29]	Matrix: AUDMA (Aromatic Urethane Dimethacrylate) DDDMA (1,12-Dodecane Dimethycrylate) UDMA (Urethane dimethacrylate) Fillers: Non-aglomerated/non-agregated 20 nm silica and 4–11 nm zirconia, aggregated zirconia/silica cluster (comprised of 20 nm silica and 4–11 nm zirconia particles), and ytterbium fluoride (agglomerated 100 nm particles).	10–20% <10% 1–10%	23.5/76.5
3	FC G-aenial Universal Flo [30,31]	Matrix: UDMA (Urethane dimethacrylate) Bis-EMA Dimethacrylate component (TEGDMA) Fillers: Silicon dioxide (16 nm), strontium glass (200 nm), pigments.	10–20% 5–10% 5–10%	31/69

**Table 2:** Experimental design [26].

No.	Composite Type			UC		BC		FC	
	Dimensionless Governing Factors			${\bar{Y}}_{1},\;HV$	${\bar{Y}}_{2},\;HV$	${\bar{Y}}_{1},\;HV$	${\bar{Y}}_{2},\;HV$	${\bar{Y}}_{1},\;HV$	${\bar{Y}}_{2},\;HV$
	$x_{1}$	$x_{2}$	$x_{3}$						
1	−1	−1	−1	42.0	33.5	59.1	55.8	42.4	37.9
2	1	−1	−1	52.4	42.9	61.7	60.2	50.0	46.3
3	−1	1	−1	45.9	41.1	61.8	61.1	47.5	45.5
4	1	1	−1	57.8	51.3	68.4	67.5	49.9	47.7
5	−1	−1	1	45.0	12.2	57.9	45.4	42.9	13.1
6	1	−1	1	58.9	26.1	61.7	55.3	45.0	27.0
7	−1	1	1	49.3	32.7	60.3	57.5	45.3	31.7
8	1	1	1	62.7	45.0	67.2	65.3	48.1	42.6
9	−0.1111	−1	−1	54.1	42.2	62.2	60.3	47.7	42.3
10	−0.1111	1	1	56.9	35.8	65.1	61.6	45.8	37.6
11	−1	0	−1	42.4	38.2	63.8	60.3	45.3	43.5
12	1	0	1	61.7	38.4	65.3	62.5	45.5	36.5
13	−1	−1	0	44.3	22.2	59.2	51.8	46.1	29.9
14	1	1	0	59.3	48.7	69.1	67.3	51.1	47.1

**Table 3:** Governing factors and their levels.

$\mathrm{Light}\;\mathrm{intensity}\;I\;[mW/cm^{2}]$	${\tilde{x}}_{1}$	600	1000	1500
Irradiation time t [s]	${\tilde{x}}_{2}$	20	40	60
Layer thickness d [mm]	${\tilde{x}}_{3}$	2	3	4

**Table 4:** Polynomial coefficients of the objective functions.

Composite/Objective Function		Polynomial Coefficients									
		$b_{0}$	$b_{1}$	$b_{2}$	$b_{3}$	$b_{11}$	$b_{22}$	$b_{12}$	$b_{13}$	$b_{23}$	$b_{123}$
UC	$Y_{1}$	56.266	6.4	1.53	2.002	−4.458	0	0	0	0	0
	$Y_{2}$	35.936	6.11	6.459	−6.991	0	0	0	1.189	3.083	0
BC	$Y_{1}$	64.55	2.327	2.25	−0.704	0	−1.866	1.003	0	0	0
	$Y_{2}$	59.11	3.59	4.17	−2.74	0	0	0	1.162	1.397	0
FC	$Y_{1}$	46.762	1.715	1.054	−1.326	0	0	0	−0.766	0	0.730
	$Y_{2}$	37.091	4.214	5.271	−7.769	0	0	0	1.994	3.185	0

**Table 5:** Factors influencing the photopolymerization process of resin-based dental composites.

Composite	Factors		
	Priority	Top Surface	Bottom Surface
UC Evetric	1 high	$x_{1}$—light intensity	$x_{3}$—layer thickness
	2 medium	$x_{3}$—layer thickness	$x_{2}$—curing time
	3 low	$x_{2}$—curing time	$x_{1}$—light intensity
BC Filtek One Bulk Fill Restorative	1 high	$x_{1}$—light intensity	$x_{2}$—curing time
	2 medium	$x_{2}$—curing time	$x_{1}$—light intensity
	3 low	$x_{3}$—layer thickness	$x_{3}$—layer thickness
FC G-aenial Universal Flo	1 high	$x_{1}$—light intensity	$x_{3}$—layer thickness
	2 medium	$x_{3}$—layer thickness	$x_{2}$—curing time
	3 low	$x_{2}$—curing time	$x_{1}$—light intensity

## Nomenclature

$b_{jk}$	Polynomial coefficients
$x_{i}$	Variable in coded form
${\tilde{x}}_{i}$	Variable in natural form
${\bar{Y}}_{i}$	Measured microhardness: i=1—top; i=2—bottom
$Y_{i}$	Objective function

## Abbreviations

ANOVA	Analysis of variance
BC	Bulk composite
FC	Flowable composite
LCU	Light curing unit
RBCs	Resin based composites
UC	Universal composite
